# MINIMALLY INVASIVE SURGERY FOR GASTRIC CANCER: PARADIGM SHIFT

**DOI:** 10.1590/0102-672020190001e1483

**Published:** 2019-12-20

**Authors:** Marcus Fernando Kodama Pertille RAMOS, Leandro Cardoso BARCHI, Antonio Carlos WESTON, Bruno ZILBERSTEIN

**Affiliations:** 1 Brazilian Gastric Cancer Association;; 2 Cancer Institute, Hospital das Clínicas, School of Medicine, University of São Paulo, São Paulo, SP;; 3 Santa Casa de Misericórdia of Porto Alegre, Porto Alegre, RS, Brazil

Minimally invasive laparoscopic surgery has gained popularity and has become the standard method for approaching various surgical procedures.(10) However, its use for the treatment of gastric cancer (GC) has always cast doubts on the possibility of properly performing the complex lymphadenectomy involved in these procedures.(2) As with any incorporation of a new technology in surgery, initial reports of its use involved only retrospective case series of selected patients. This large selection bias has always led to questions about the actual effectiveness of the method and its generalizability.

Despite this undeniable limitation of initial studies, preliminary results from other centers, as well as our own experience, showed us that the laparoscopic approach to gastric tumors was feasible.(3) The advantages already described of the laparoscopic method, such as lower postoperative pain, earlier recovery and discharge, were maintained. The surgical time was longer, but the most important fact of these initial studies was the certainty that the postoperative complications were equivalent and the early surgical oncological parameters, such as lymph nodes count and surgical margins did not change. These results encouraged us already in 2016 to question whether it was time for a paradigm shift from laparotomic surgery to minimally invasive laparoscopic surgery.(1) Perhaps, at that time we relied on non-ideal and retrospective evidence giving the impression that it might be wishful thinking. However, the results presented in subsequent years of large prospective randomized trials eventually confirmed our initial impression. 

Undoubtedly a large number of studies have been published on the subject in recent years. Critical analysis of these studies is important, and details should not be confused so that wrong conclusions are not made. Some points should always be considered when analyzing these studies of the use of minimally invasive surgery in the GC: 1) What type of tumor was evaluated: early or advanced? 2) Was the surgical resection distal or total? 3) Was the study prospective randomized or were the cases selected? 4) Are the analyzed outcomes early related to long-term surgical or cancer complications? 5) Was the study of superiority or non-inferiority? 6) Does the data reproduce the reality of my practice? 

The major milestone in the use of laparoscopic surgery in the treatment of GC was the publication of the results of the South Korean study KLASS-01.(8) This non-inferiority study compared laparoscopic with laparotomic surgery for the treatment of early distal tumors. The laparoscopic method presented lower occurrence of surgical complications related to surgical wound without inferiority in relation to disease free survival in three years. Subsequently the Japanese study JCOG0912 confirmed the equivalence of early surgical results but late oncologic results have not yet been released.(7) Since then, laparoscopic surgery has been considered the standard for resection of early distal gastric tumors. Still evaluating early but proximal tumors, two studies - KLASS-03 and JCOG1401 - have already shown similar early surgical results and it is expected that long-term oncological results will be publish soon.

Laparoscopic surgery in advanced tumors has always raised greater concern regarding the possibility of peritoneal dissemination by manipulation of tumors affecting the serosa as well as the possibility of incomplete lymphadenectomy in cases with high likelihood of lymph node involvement. Preliminary surgical results from three studies - JLSSG0901, KLASS-02 and CLASS-01 - did not show any alteration in the number of early surgical complications.(5,9,11) Analysis of early oncological criteria such as surgical margins, number of resected lymph nodes and final pathological staging did not differ between groups either. However, long-term oncological results were eagerly awaited. Fortunately, during the last International Gastric Cancer Congress held in Prague in May 2019, the authors of the South Korean study KLASS-02 and Chinese CLASS-01 presented its long-term cancer results. Both demonstrated non-inferiority of laparoscopic surgery in relation to disease free survival in three years. Shortly thereafter, the CLASS-01 study was fully published in JAMA confirming the preliminary non-inferiority data presented for laparoscopic surgery.(11) Thus, we can also state that laparoscopic surgery can be employed to treat advanced distal gastric tumors. With regard to advanced proximal tumors, South Koreans started in 2018 the KLASS-06 study, but no data are available at this time. 

The question whether oriental data can be extrapolated to Western patients and surgeons is always pertinent. In this context the Dutch study LOGICA serves as a bridge between the two extremes.(4) At the 2019 International Gastric Cancer Congress - Prague, we also had the opportunity to evaluate the initial surgical data from this multicenter study involving 10 Dutch centers and 210 patients. Preliminary data presented did not show any difference between the techniques, but these results have not yet been published. 

Unfortunately, the large number of patients and funding required in these studies makes it difficult to perform in our country. Therefore, the follow-up of the results of these large international studies is essential to be updated with the latest evidence. If we do not have prospective randomized data, the critical analysis of our own results with the publication of case series is fundamental for the quality control of the technique and to verify if its generalization is possible in our country.(6) This year the Brazilian Gastric Cancer Association celebrated its 20^th^ anniversary. To celebrate this date, a commemorative day was held in Porto Alegre, Brazil, in August, when we had the opportunity to discuss these new studies. If in 2016 we did not have the best scientific evidence to make the transition to the minimally invasive approach, recent data released today supports our initial impression. Thus, considering that most tumors in our country are still distal, we believe that increasingly minimally invasive surgery will become the standard in the surgical treatment of GC.

## References

[1] Barchi LC, Jacob CE, Bresciani CJ, Yagi OK, Mucerino DR, Lopasso FP (2016). Minimally Invasive Surgery for Gastric Cancer: Time to Change the Paradigm. Arquivos brasileiros de cirurgia digestiva : ABCD = Brazilian archives of digestive surgery.

[2] Barchi LC, Ramos MFKP, Dias AR, YAGI OK, Ribeiro U, Zilberstein B, Cecconello I (2019). Total Omentectomy: Is it always necessary. Arquivos brasileiros de cirurgia digestiva : ABCD = Brazilian archives of digestive surgery.

[3] Dias AR, Jacob CE, Ramos M, Pereira MA, Szor DJ, Yagi OK (2019). Laparoscopic D2 Gastrectomy for Gastric Cancer: Mid-Term Results and Current Evidence. Journal of laparoendoscopic & advanced surgical techniques Part A.

[4] Haverkamp L, Brenkman HJ, Seesing MF, Gisbertz SS, van Berge Henegouwen MI, Luyer MD (2015). Laparoscopic versus open gastrectomy for gastric cancer, a multicenter prospectively randomized controlled trial (LOGICA-trial). BMC cancer.

[5] Inaki N, Etoh T, Ohyama T, Uchiyama K, Katada N, Koeda K (2015). A Multi-institutional, Prospective, Phase II Feasibility Study of Laparoscopy-Assisted Distal Gastrectomy with D2 Lymph Node Dissection for Locally Advanced Gastric Cancer (JLSSG0901). World journal of surgery.

[6] Kassab P, da Costa WL, Jacob CE, Cordts RM, Castro OAP, Barchi LC (2017). Minimally invasive surgery for gastric cancer in Brazil: current status and perspectives-a report from the Brazilian Laparoscopic Oncologic Gastrectomy Group (BLOGG). Translational gastroenterology and hepatology.

[7] Katai H, Mizusawa J, Katayama H, Takagi M, Yoshikawa T, Fukagawa T (2017). Short-term surgical outcomes from a phase III study of laparoscopy-assisted versus open distal gastrectomy with nodal dissection for clinical stage IA/IB gastric cancer: Japan Clinical Oncology Group Study JCOG0912. Gastric cancer : official journal of the International Gastric Cancer Association and the Japanese Gastric Cancer Association.

[8] Kim W, Kim HH, Han SU, Kim MC, Hyung WJ, Ryu SW (2016). Decreased Morbidity of Laparoscopic Distal Gastrectomy Compared With Open Distal Gastrectomy for Stage I Gastric Cancer: Short-term Outcomes From a Multicenter Randomized Controlled Trial (KLASS-01). Annals of surgery.

[9] Lee HJ, Hyung WJ, Yang HK, Han SU, Park YK, An JY (2019). Short-term Outcomes of a Multicenter Randomized Controlled Trial Comparing Laparoscopic Distal Gastrectomy With D2 Lymphadenectomy to Open Distal Gastrectomy for Locally Advanced Gastric Cancer (KLASS-02-RCT). Annals of surgery.

[10] Ramos MFKP, Pereira MA, Charruf AZ, Dias AR, Castria TB, Barchi LC (2019). Conversion Therapy for Gastric Cancer: Expanding the Treatment Possibilities. Arquivos brasileiros de cirurgia digestiva : ABCD = Brazilian archives of digestive surgery.

[11] Yu J, Huang C, Sun Y, Su X, Cao H, Hu J (2019). Effect of Laparoscopic vs Open Distal Gastrectomy on 3-Year Disease-Free Survival in Patients With Locally Advanced Gastric Cancer: The CLASS-01 Randomized Clinical Trial. Jama.

